# Integration of ground survey and remote sensing derived data: Producing robust indicators of habitat extent and condition

**DOI:** 10.1002/ece3.5376

**Published:** 2019-06-20

**Authors:** Peter A. Henrys, Susan G. Jarvis

**Affiliations:** ^1^ NERC Centre for Ecology and Hydrology Lancaster Environment Centre Lancaster UK

**Keywords:** Bayesian model calibration, data integration, field survey, Great Britain, peatland, remote sensing

## Abstract

The availability of suitable habitat is a key predictor of the changing status of biodiversity. Quantifying habitat availability over large spatial scales is, however, challenging. Although remote sensing techniques have high spatial coverage, there is uncertainty associated with these estimates due to errors in classification. Alternatively, the extent of habitats can be estimated from ground‐based field survey. Financial and logistical constraints mean that on‐the‐ground surveys have much lower coverage, but they can produce much higher quality estimates of habitat extent in the areas that are surveyed. Here, we demonstrate a new combined model which uses both types of data to produce unified national estimates of the extent of four key habitats across Great Britain based on Countryside Survey and Land Cover Map. This approach considers that the true proportion of habitat per km^2^ (*Z_i_*) is unobserved, but both ground survey and remote sensing can be used to estimate *Z_i_*. The model allows the relationship between remote sensing data and *Z_i_* to be spatially biased while ground survey is assumed to be unbiased. Taking a statistical model‐based approach to integrating field survey and remote sensing data allows for information on bias and precision to be captured and propagated such that estimates produced and parameters estimated are robust and interpretable. A simulation study shows that the combined model should perform best when error in the ground survey data is low. We use repeat surveys to parameterize the variance of ground survey data and demonstrate that error in this data source is small. The model produced revised national estimates of broadleaved woodland, arable land, bog, and fen, marsh and swamp extent across Britain in 2007.

## 1 INTRODUCTION

Addressing biodiversity loss is widely identified as a major environmental challenge of the 21st century (CBD, 2011). Of the many factors identified in the literature contributing to the changing status of biodiversity, a commonly occurring theme is the availability of sufficient habitat. Studies have shown that availability of habitat can significantly affect species' population trends (Andren, 1994; Warren et al., 2001), range expansion rates (Hill et al., 2001; Wilson, Davies, & Thomas, 2010), and survival success (Krauss, Steffan‐Dewenter, & Tscharntke, 2003). The quality and connectivity of the habitat also play crucial roles in the variation and change of species' populations (Didham, Tylianakis, Gemmell, Rand, & Ewers, 2007; Lindborg & Eriksson, 2004). Hodgson, Moilanen, Wintle, and Thomas (2011) provide an excellent overview of published studies relating to the impact of habitat area, habitat quality, and spatial connectivity on species. Habitats are also important features within their own right as they are a key natural capital asset which can provide multiple benefits relating to food, clean air, recreation, clean water, and hazard protection (Mace, Hails, Cryle, Harlow, & Clarke, 2015). Understanding the spatial extent and distribution of any particular habitat is therefore important not only for understanding habitat fragmentation and loss, but also to anticipate any potential impact on species' distribution and abundance and to effectively manage natural resources (Kareiva & Wennergren, 1995).

Accurately estimating habitat cover over large spatial scales is challenging. Two main approaches exist to extrapolate from a subsample of the area surveyed on the ground or to use remote sensing from satellites which can provide full census coverage. On‐the‐ground data may arise from surveys which predominantly focus on habitat monitoring (e.g., NCC English Field Unit, 1990) or that record habitat information as an additional measure as part of a wider environmental assessment (Norton et al., 2012) or focused taxonomic study (Baker & Gleed‐Owen, 2007; Risely et al., 2011). However, for large regions extensive, fully representative, ground‐based field survey is often impractical or too expensive. Therefore, national estimates of habitat cover from on‐the‐ground surveys are derived by statistical extrapolation (Hamre, Domaas, Austad, & Rydgren, 2007; Howard, Watkins, Clarke, Barnett, & Stark, 2003; Martino & Fritz, 2008).

Habitat coverage can also be estimated from remote sensing via satellites or unmanned aerial vehicles (UAVs) operating in the red, near or mid‐infrared spectral bands (Carrasco, O'Neil, Morton, & Rowland, 2019; Cruzan et al., 2016; Debinski, Kindscher, & Jakubauskas, 1999; Morton et al., 2011; Stratoulias, Balzter, Sykioti, Zlinszky, & Tóth, 2015). Remote sensing from satellites has an advantage over ground‐based field surveys in that that the spatial distribution as well as the total area can be estimated thanks to its census coverage. However, remote sensing does not provide a direct measurement of habitat cover. Image pixels must be classified as belonging to a certain habitat using some classification algorithm on the raw spectral frequencies. The accuracy of the classification algorithm is dependent on the availability of high‐quality training data, and all algorithms will introduce some degree of error or uncertainty. In addition, bias may be introduced if, for example, there is any spatial variation in the relationship between optical frequencies and land cover due to climatic gradients, for example, which is not captured in the training data. The availability of sufficient image data can also be severely hampered by cloud cover.

Broadly speaking, the two available data sources to estimate national habitat cover therefore fall into the categories of high accuracy, unbiased but low‐coverage information (on‐the‐ground assessment) and lower accuracy, potentially biased high‐coverage information (remote sensing). To provide robust estimates of habitat extent, it would therefore be optimal to combine the data from both sources. Here, we present an approach to integrate data from remote sensing and ground survey within a single unified model to produce estimates of habitat extent at a national level for Great Britain. The approach presented provides a method to estimate the true, unobserved, habitat extent using multiple data sources, while quantifying and accounting for bias and variance in the data. We use the model to estimate the areal extent of a number of key broad habitats across Britain.

## 2 MATERIALS AND METHODS

### 2.1 Data

**Figure 1:**
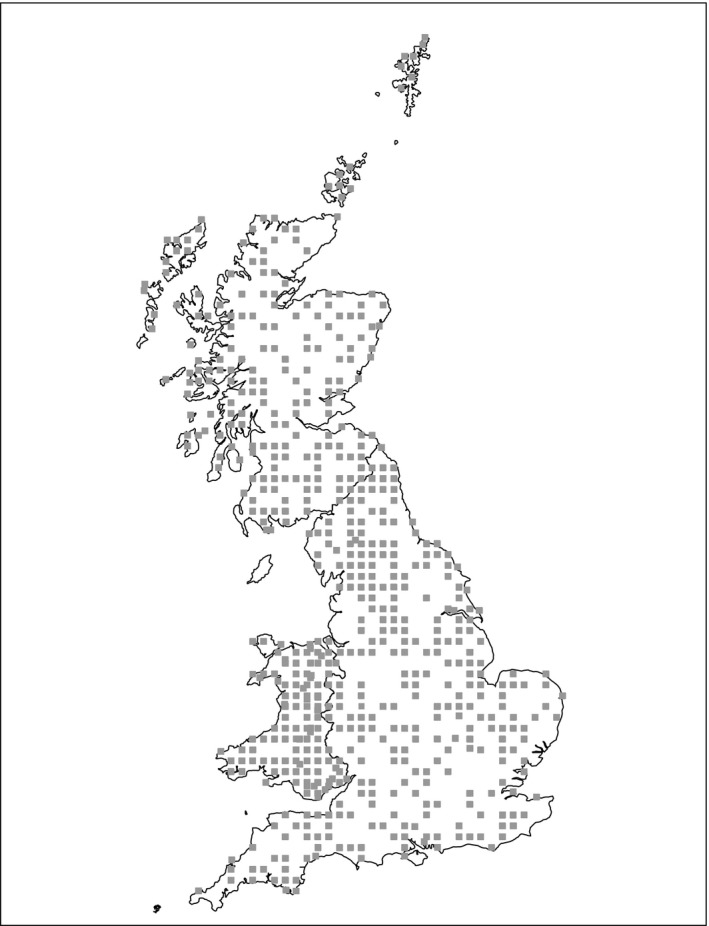
Locations of 1 km × 1 km CS squares surveyed in 2007

Ground survey data came from the Countryside Survey (CS) of Great Britain (Brown et al., 2016; Norton et al., 2012), which is a nationwide assessment of stock and change of vegetation, soil, habitats, landscape features, and freshwaters. The survey samples 1 km × 1 km squares across Britain within which all habitats and features are accurately mapped (according to a minimal mappable unit of 20 m^2^) and described. Survey squares are sampled randomly within 45 strata known as land classes to ensure representative coverage of the environmental conditions across GB. Figure 1 shows the locations of the 591 squares surveyed in 2007, the most recent survey to date. Every polygon within the square is assigned to a habitat type based on the UK's Joint Nature Conservation Committee's broad and priority habitat classifications (Jackson, 2000). Total habitat areas are then obtained by estimating the proportion of each habitat type within each of the sampling strata (Bunce, Barr, Clarke, Howard, & Lane, 1996; Howard et al., 2003). A generalized linear mixed model (McCulloch & Neuhaus, 2005) approach is used to estimate the average proportion of each 1 km square covered by each specific habitat in each stratum, accounting for temporal correlation across the repeated surveys by inclusion of an AR(1) component in the model capturing correlation across the repeats, which are approximately every 10 years. The total area covered is then calculated by multiplying this estimate by the area of the respective stratum to produce a total estimated area of habitat per land class. Summing over all land classes provides a total area over Great Britain. Confidence intervals around these estimates are obtained using a bootstrap approach (Efron & Tibshirani, 1994) of resampling squares, with replacement, within strata.

The remote sensing product used was the 2007 Land Cover Map (LCM) of Great Britain (Morton et al., 2011,2014), which is a classification of satellite imagery compiled from Landsat, IRS, and SPOT into different habitat categories using maximum‐likelihood classification techniques. The satellite data are integrated with the Ordnance Survey master Map spatial framework (OS MasterMap Topography Layer, 2007) to provide a field parcel level, down to 25 m resolution, habitat classification. Composite satellite images across different temporal periods are used in order to provide full coverage of GB, though due to cloud cover and image availability, different regions are based on different composites. The maximum‐likelihood classifier is based on training data obtained from an independent field survey campaign providing reference points and associated land cover data. Reference points were specifically chosen to ensure that all land cover types, including rarer ones, were adequately covered. Areal extent of any individual habitat is simply calculated by summing parcel areas within each class. No confidence intervals are currently provided on these estimates. While there is a more recent version of the LCM, based on Sentinel 2 data from 2015 (Rowland et al., 2017), the 2007 product was used here as it aligns with the temporal period covered by the CS field survey.

Both the CS and the LCM therefore provide estimates of the areal extent of each broad habitat in Great Britain in 2007. Table 1 shows a comparison between the estimated extents for the two schemes. The reported total areas can vary dramatically between different habitat categories with no consistent difference between under‐ or overestimation of one scheme relative to the other. The two schemes also provide estimates of the proportion of individual 1 km squares covered by each habitat type. For LCM, this covers every 1 km square in Great Britain, whereas for CS, proportions are only available from the 591 sampled squares. Both approaches have some element of uncertainty associated with them. For the CS ground‐based estimates, this uncertainty is mainly due to the upscaling from sampled squares to large spatial regions, which one may think of as sampling uncertainty. In addition to this, the sampling uncertainty is dependent on representative observations and any bias that may exist in the sample will potentially increase the overall uncertainty. Here, the CS sample is considered representative of different environmental conditions across GB due to the stratification by Land Class and we therefore assume this dataset is unbiased in the model. The LCM remote sensing‐based estimates contain uncertainty due to the classification of satellite imagery into habitat classes. We can think of this as model uncertainty. Neither estimate therefore perfectly reflects the true extent of GB habitats. Estimation of this underlying true state is the aim of the integrated modeling approach described below.

**Table 1:** Reported estimates of total habitat area (in 000s ha) from the Countryside Survey (CS) and Land Cover Map (LCM)

Broad habitat	CS	LCM
Broadleaved, Mixed and Yew Woodland	1,406	1,319
Coniferous Woodland	1,319	1,440
Arable and Horticulture	4,608	6,219
Improved Grassland	4,494	5,528
Neutral Grassland	2,176	1,414
Calcareous Grassland	57	37
Dwarf Shrub Heath	1,343	2,039
Fen, Marsh, and Swamp	392	10
Bog	2,232	1,005

### 2.2 Model

Let us focus on estimating the habitat extent of one particular habitat across Britain. We denote $Z_{i}$ as the true proportion of square i covered by the habitat in question, where i=1,⋯,233286 represents each 1 km by 1 km square in GB. We also take $Q_{i}$ to be the estimated proportion of square i classified as that same habitat according to remote sensing data, in this case LCM, and $Y_{i}$ as proportion recorded from ground survey data, taken here to be from CS. Note that $Y_{i}$ is sparsely populated due to the sampling regime of CS. We assume that the ground survey data are an unbiased estimate of the true proportion with some measurement error. In practice, this error could be due to either misclassification of the habitat or to misspecification of the parcel boundaries within the square. We allow for the possibility of bias in the remote sensing data as evidence provided in Morton et al. (2011) suggests that this is possible due to the image classification on the spectral signal. This bias may not necessarily be spatially homogeneous due to the use of different composite images used in different regions and therefore we allow for spatially varying bias. We assume

(1) $$Y_{i}∼N^{0}\left(Z_{i},\sigma^{2}\right)$$

(2) $$Q_{i}∼N^{0}\left(\alpha_{i}+\beta_{i}Z_{i},\tau^{2}\right),$$

where *N* ^0^ represents the normal distribution truncated at 0 and

$$\alpha_{i}=\delta_{1}+\theta_{1}{\text{Nrth}}_{i}+\theta_{2}{\text{East}}_{i},\;\beta_{i}=\delta_{2}+\theta_{3}{\text{Nrth}}_{i}+\theta_{4}{\text{East}}_{i},$$

and we are interested in estimating the true proportion *Z_i_*, shared across both models. The bias in the LCM estimates is a spatially varying function of the true proportion as *α* and *β* depend on the spatial location of square *i* (easting, East, and northing, Nrth). The parameters *α* and *β* represent the bias irrespective of and dependent on the true habitat patch size *Z_i_*, respectively, while *δ*_1_ and *δ*_2_ represent the constant element of the bias and *θ*_1,…4_ the spatial influence. Here, we use the truncated normal distribution as a reasonable alternative to a binomial or beta distribution due to the explicit specification of the variance parameters and the intuitive understanding, and identifiability, of all model terms. This would not be the case when using beta distribution, for example. The approximation is sufficient as the distribution is conditional on the true proportion for the given square *Z_i_* and sample size is typically large.

To estimate the parameters in the above model, as well as the unknown *Z_i_*, we use an MCMC approach embedding the model within a Bayesian framework. With uninformative priors, the model can be too flexible and the MCMC chain can struggle to converge to a consistent parameter set. This is due to the trade‐off that would exist between the model assuming confidence in the *Q_i_* or alternatively *Y_i_* and parameter estimates varying accordingly. This is often referred to as being nonidentifiable. Additional information is therefore needed to set informative priors or to constrain model parameters. Within the CS ground survey, an extensive quality assurance (QA) exercise is coordinated that involves a significant proportion of squares independently resurveyed by a different field team with similar levels of expertise (Norton, Scholefield, Maskell, & Smart, 2007). The resurvey takes place immediately following the initial survey so that features should be identical between the two visits. This extra information therefore provides us with an estimate of the variance (*σ* ^2^) associated with the ground survey estimates *Y_i_*. An informative prior can therefore be placed on *σ* which enables the parameters to be identifiable and the MCMC algorithm to reach convergence due to the reduction in induced flexibility. The overall model can hence be seen as a specific case of Bayesian model calibration (e.g., Van Oijen, Rougier, & Smith, 2005). The model was fitted using the JAGS software (Plummer, 2003) called via R (R Core Team, 2016) using the rjags library (Plummer, 2016). This uses a form of Gibbs sampling algorithm whereby an adaptive rejection Metropolis sampler is the main workhorse.

### 2.3 Simulation study

To evaluate the potential of a combined approach, utilizing both the ground survey and the remote sensing data, and to understand its accuracy, we conducted a simulation study. The purpose of the simulation study was to simulate hypothetical data where the total habitat extent was known and could be compared against estimates from the proposed model and estimates from either the ground survey sample‐based approach only or the remote sensing census approach only. To generate the simulated data, we first simulated some true proportions of habitat cover per cell on a 100 by 100 grid according to a truncated normal distribution with mean given by a single random draw from a uniform distribution (0, 0.2) and variance given by a single random draw from a uniform distribution (0.01, 0.05). Full coverage estimates, representing the remote sensing data, are then generated from these true values with some standard deviation, corresponding to τin Equation 2, governed by a single draw from a uniform distribution (0, 0.15). A value for the systematic bias, α in Equation 2, was taken from a uniform distribution (−0.02, 0.02) and added to the simulated estimate. This was hence taken to be a constant value that did not vary spatially, effectively taking $\theta_{1}$ and $\theta_{2}$ to be equal to 0. The bias represented by β was ignored, and therefore, β was set equal to 1. Therefore, the simulated remote sensing data included some error (τ) and also some constant bias (α), but did not include bias which varied with the true habitat value (i.e., β was set to 1). Sample estimates, representing a typical ground survey, are then also generated for a subset of 25 grid cells according to the true value with standard deviation (corresponding to σ in Equation 1) drawn from a uniform (0, 0.02) distribution. Therefore, the simulated ground survey was unbiased, but included a small amount of error. This process was repeated 1,000 times to generate 1,000 estimated datasets representing both the census coverage remote sensing data and the sample‐based ground survey data. Due to the drawing of parameters from uniform distributions at each iteration, performance under a range of different distributional assumptions is incorporated within the simulated data rather than from fixed parameterizations at each iteration.

In addition to this, the whole process was repeated once more with the variance on the ground survey estimates, σ, taken as a sample from a uniform distribution on (0, 0.2) to investigate how this increased uncertainty would impact on the conclusions of the combined model. This simulation would therefore allow us to determine the effectiveness of a combined approach when data from both sources are highly variable. Each of the simulated datasets was analyzed using the same model as presented in the previous section to compare the estimated results to the truth.

## 3 RESULTS

We used the proposed model to estimate the total coverage of four habitats (broadleaved woodland, bog, arable, and fen, marsh and swamp) across Britain. The QA data available from the CS suggested small variation between the two independent repeat visits to the same square across all habitats (Norton et al., 2007). This information was used to provide informative uniform priors for all four habitats for *σ*. As this variation in this standard deviation parameter was known to be relatively small across all habitats, while *τ* remains highly flexible in its specification, more “weight” is effectively given to the CS data within the model framework.

Results from the model show bias in the LCM data across all habitats, which appears to have a significant spatial effect. This is demonstrated by the parameter estimates in Table 2 where the credible intervals obtained from the posterior distribution do not contain 0 for at least one of the θ parameters across all four habitats. Maps of α and β shown in Appendix S1 provide a visualization of the spatial bias for each broad habitat. They differ in each case both in terms of the effect size and the main direction of the gradient, highlighting the importance of this flexible spatial effect in the model. This spatial effect may be a result of the use of different composite images across the region, due to cloud cover, resulting in spatially explicit bias or where the timing of images used differs across the region. The CS results are unbiased and have low variation demonstrated by the estimates (*σ* ^2^) shown in Table 2, which are relatively low for all habitats. This is not surprising as the model imposes that the CS data are unbiased and an informative and small prior for σ has been used. The variance related to the LCM data (*τ* ^2^) is generally much lower with the exception of fen, marsh, and swamp (FMS). In this case, the variance estimates are extremely small and the *R* ^2^ values showing the relationship between the CS and LCM data, shown in Table 3, are extremely low. In this case, the model did not converge most likely due to the large discrepancy in the raw data between the two data sources and as such the parameter estimates should not be trusted. This highlights a potential issue with the joint modeling approach when there is little agreement between individual data sources.

**Table 2:** Parameter estimates from joint models fitted to broadleaved woodland, bog, arable and fen, marsh, and swamp

	*δ*_1_	*δ*_2_	*θ*_1_	*θ*_2_	*θ*_3_	*θ*_4_	*σ* ^2^	*τ* ^2^
Broadleaved woodland								
50%	−0.340	0.475	5.34E−07	3.18E−07	2.40E−07	3.86E−07	3.04	17.54
2.50%	−1.150	0.383	−1.03E−06	−6.64E−07	6.80E−08	2.23E−07	3.94	19.61
97.50%	0.435	0.571	2.25E−06	8.88E−07	4.04E−07	4.81E−07	2.48	15.63
Bog								
50%	1.019	1.242	−5.23E−06	3.42E−06	−3.63E−06	−1.28E−07	3.56	111.11
2.50%	0.213	0.070	−7.45E−06	2.05E−06	−4.26E−06	−6.24E−07	4.00	142.86
97.50%	1.863	1.642	−3.50E−06	4.78E−06	−1.10E−07	4.14E−07	2.70	41.67
Arable								
50%	−0.424	0.467	7.67E−07	4.43E−07	7.61E−07	2.22E−07	2.65	90.91
2.50%	−1.552	0.412	−3.35E−06	−1.30E−06	6.97E−07	1.42E−07	3.83	100.00
97.50%	1.466	0.515	3.25E−06	1.56E−06	8.87E−07	2.66E−07	1.87	76.92
Fen, marsh, and swamp								
50%	0.616	−0.338	−1.29E−06	−2.13E−08	2.25E−06	−7.02E−07	10.20	0.01
2.50%	0.366	−0.552	−1.87E−06	−3.68E−07	1.55E−06	−1.53E−06	11.36	0.03
97.50%	0.847	−0.084	−1.06E−06	2.41E−07	3.11E−06	6.34E−07	9.17	0.01

Note

Shown are the 50th percentile and 2.5th and 97.5th percentiles from the taken from the posterior distributions for each parameter.

**Table 3:** Estimated total area of each broad habitat across Britain (in 000s ha) together with differences from previously reported estimates from CS and LCM as shown in Table 1 and the *R* ^2^ value of the relationship between the CS square values and corresponding LCM values

Habitat	Total Est (000s ha)	Difference from CS estimate	Difference from LCM estimate	*R* ^2^ between CS and LCM
Broadleaved woodland	1,176	−230	−143	0.475
Bog	1,025	−1,207	20	0.143
Arable	5,408	800	−811	0.857
Fen, marsh, and swamp	193	−199	183	0.000

Estimates of the total extent of each broad habitat across Britain are shown in Table 3. The revised estimates for each of the four habitats seem sensible and consistent with the previously reported estimates shown in Table 1, though care should be noted with the FMS estimates as mentioned. It is also worth noting that the revised estimate for broadleaved woodland is lower than both the reported CS and LCM estimates. Though one might intuitively assume a joint estimate would fall between the two, there are no reason and no imposition within the model that the revised estimate should. The combined approach has the advantage of utilizing the high‐quality CS data to achieve unbiased estimates, while maintaining the census coverage offered by LCM.

**Figure 2:**
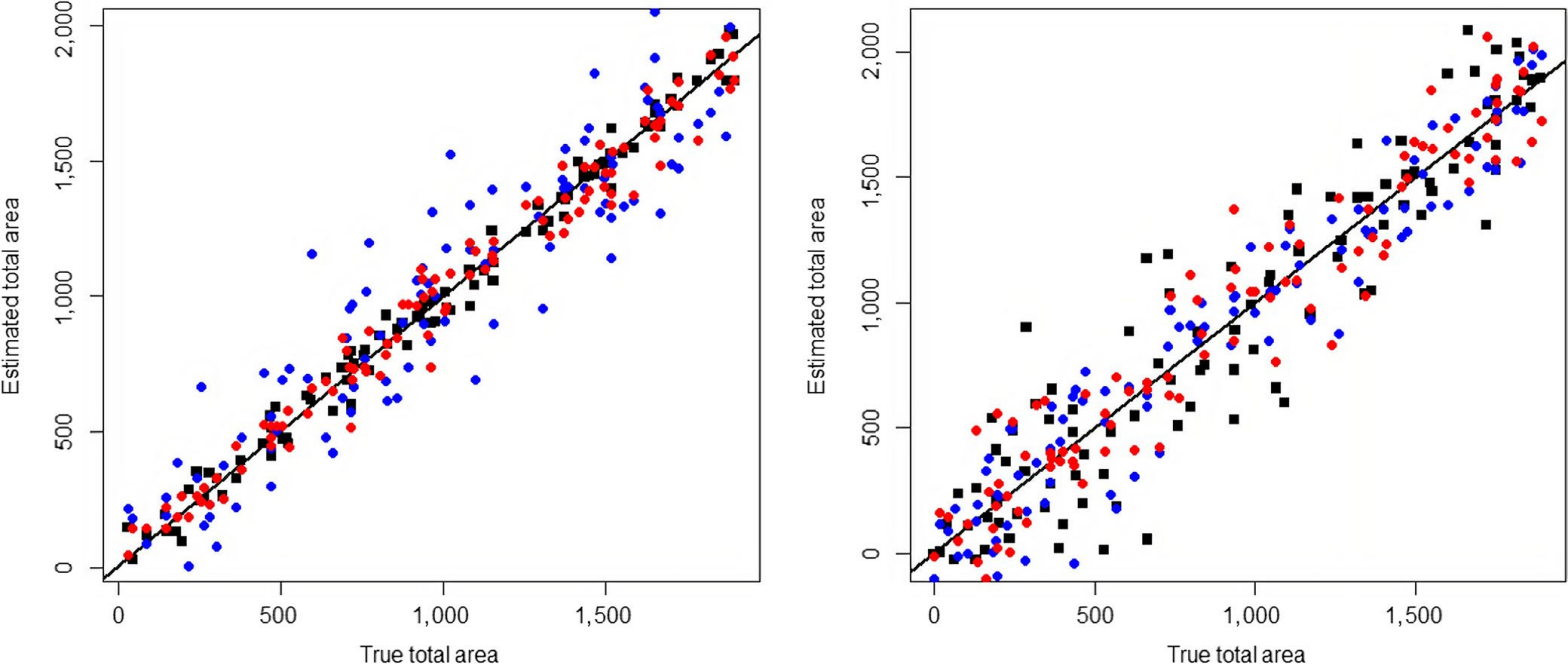
Results from simulation study showing the observed versus estimated total area for the 1,000 simulated datasets. Left‐hand plot shows simulation when the field survey error is low and the right‐hand plots when the error is large. Black represents estimates form combined model, blue from LCM‐type census coverage, and red from extrapolated field survey

Results from the simulation study are shown in Figure 2 where both plots show the estimated versus true total areas based on LCM‐type census coverage only (blue), ground survey samples only (red), and the combined model (black). The left‐hand plot shows results when error in the ground survey data is low and the right plot when the error can be large. In the left‐hand plot, the combined model performs best with a greater predictive accuracy of the truth confirmed by root–mean‐square error (*RMSE*) values, which represent error in estimated coverage, shown in Table 4. Whereas when the error in the ground data can be high (right‐hand plot, Figure 2), the combined model performs relatively poorly and the LCM‐type census is optimal, also confirmed by *RMSE* (Table 4). This highlights the issue of estimating a latent variable, in this case the true proportion Z, from two sources with high variation in each. Parameters within the model, shown in Equations 1 and 2, can be difficult to estimate, unidentifiable, and may fail to converge.

**Table 4:** Root‐mean‐square error values for the different approaches to estimating habitat coverage compared to the known true coverage

Standard deviation of ground survey proportions	Combined model	Remote sensing census only	Ground survey only
Uniform(0, 0.02)	82.50	183.59	137.97
Uniform(0, 0.2)	447.02	191.66	258.11

Note

This is shown for when the variation in the ground survey data is low (top row) and high (bottom line).

## 4 DISCUSSION

We have presented a method to jointly analyze data on habitat coverage from two distinct sources, which we took to be ground‐based field survey and remote sensing derived data, within the same framework to estimate habitat extents across large regions. The simulation study showed that the proposed method performs well when the uncertainty in the ground‐based data is low. It offers a significant improvement over using each data source independently and has the potential to overcome uncertainty issues present in each of the data sources. In the example presented, the variability in ground‐based data was known to be low based on QA data collected alongside the main ground survey data.

The simulation study also showed that when there was a high degree of uncertainty in the ground survey data, then there was no benefit in combining. This is because when there are disagreement and large variation in the two data sources, the model has no way of knowing which is “correct” and can essentially calibrate the data most closely reflecting the truth using the other dataset and hence induce bias. What is also clear is that when there is high uncertainty in the data collected and this is ignored, the inference can be far worse. The simulation study therefore highlights the importance of QA procedures to provide some understanding of the potential uncertainty associated with data collection so that informed decisions can be made about when to use integrated modeling.

The model used is a particular form of latent variable model, where the unknown in this case represents the true coverage proportion of habitat per kilometer square. Latent variable models can often suffer issues with convergence and identifiability, as we have seen in some cases here. In such cases, informative priors can overcome convergence issues. For estimating habitat extents, information from the QA survey was used to provide an informative prior on the error in the CS data which provided enough information for the model to converge. Therefore, a robust QA procedure that can ascertain variability in observations can not only help to determine whether integrated modeling is appropriate, but also provide information for the prior distributions used in the modeling approach.

Effective QA exercises, such as that undertaken in the CS, may be more complex to apply to remote sensing data but should be seen as essential for ground‐based survey. This level of QA should be perfectly possible in citizen science schemes as well as professional‐based survey as the only requirement is for independent resurvey of a random subset of sites. The extra information that such exercises can provide with respect to quantifying uncertainty (Scott & Hallam, 2003) can be critical for robust methods and models such as that presented here.

When there is disagreement between data sources, this could be due to a number of reasons and it is important to recognize the limitations of each dataset and potential ecological differences prior to a joint modeling exercise. For example, the case study showed that there was no agreement (*R* ^2^ < 0.01) between FMS coverage between CS and LCM. This is a known issue as FMS can be comprised of different land cover types and typically occurs in small patches that fall below the minimum mappable unit of the remotely sensed LCM (0.5 ha). Therefore, there may be a difference in the ecological interpretation of the FMS habitat category between the two datasets, meaning that an integrated model may not be appropriate for estimating the extent of this habitat. Similarly, the coverage of bog across CS and LCM showed limited agreement (*R* ^2^ = 0.143) which may reflect differences in definitions of bog habitats between the two datasets as well as challenges in identification of this habitat type via remote sensing.

The model presented has application beyond habitat extent as remote sensing data are increasingly being used to look at additional environmental indicators (Lawley, Lewis, Clarke, & Ostendorf, 2016; O'Connor et al., 2015; Pettorelli, Safi, & Turner, 2014). In such circumstances, the model presented could offer significant advantages by combining the remote sensing data with ground‐based field survey data collected as part of a citizen science or professional survey campaign. Typically, such data are used alongside the remote sensing data to provide a simple scaling or conversion metric that is not spatially explicit nor is the uncertainty in this propagated through to the end result (Lawley et al., 2016; Tebbs, Remedios, Avery, Rowland, & Harper, 2015; Wanders, Karssenberg, Roo, Jong, & Bierkens, 2014). The model we have presented would enable a spatially explicit calibration of the remote sensing data, while accounting for uncertainty, using detailed ground‐based observation that could significantly improve estimation and inference of key environmental indicators. In addition to this, the model itself could also be extended to incorporate a temporally explicit component. Estimating land cover change is known to be challenging and can often have a high degree of uncertainty (Prestele et al., 2016), potentially due to changing quality and availability of satellite data. Therefore, using an approach similar to that presented here to account for the changing uncertainty, calibrated alongside ground‐based data could enable robust estimation of land cover change metrics. There is also the potential to evolve the model into a joint distribution modeling framework (e.g., Pollock et al., 2014) such that data at different scales or sampled at different locations could be incorporated in the same model and analyzed together. While this is possible, building on the approach presented here, we see this as a nontrivial exercise and we would expect issues around convergence and identifiability.

In the model presented, we have used the truncated normal distribution, which for the particular example was shown to be appropriate. However, there are obvious circumstances whereby this distribution would not be sufficient and alternatives could and should be used. In such cases, the truncated normal could be reasonably exchanged for a beta, negative binomial or Tweedie distribution, for example. This may require some reparameterization of the model to relate mean and variance parameters to the respective scale and shape parameters and additional constraints added to ensure proper distributions. Where reasonable to do so, use of the truncated normal has the advantage of intuitive parameterization, reasonable approximation, and fast computation (Bhattacharya & Rao, 2010).

There is also potential to extend the model beyond consideration of two data sources to multiple data sources. This would be reasonably straightforward to do, merely introducing another component into the model beyond Equations 1 and 2. Additional data would have the potential of increasing the precision of the joint model estimates further, leading to more accurate estimates of broad habitat extent way beyond any assessment made from individual sources. However, there is also the possibility that model parameters are harder to estimate and identifiability is a greater issue. As with the two data source case presented, there is a trade‐off based on agreement across data sources, variability in each bias. Depending on the agreement, the level of uncertainty acceptable for convergence and model performance within each specific data source will vary. With the increase in citizen science data and opportunistic data, there is a growing volume of data that is potentially information‐rich but has high variability. For incorporation into the model framework presented here, we stress the importance of some QA exercise and the value of exploratory analysis to determine agreement and overlap across all data sources.

Overall, we therefore believe that the approach presented has large potential for improving the estimates of status and trend of key environmental indicators over large regions.

## CONFLICT OF INTEREST

None declared.

## AUTHOR CONTRIBUTIONS

Both PAH and SGJ conceived the study, PAH conducted the analyses, and both PAH and SGJ interpreted the data and wrote the manuscript.

## Data Availability

Countryside Survey 2007 habitat mapping data: https://doi.org/10.5285/bf189c57-61eb-4339-a7b3-d2e81fdde28d. Land Cover Map 2007 percentage area data: https://doi.org/10.5285/fdf8c8d3-5998-45a5-8431-7f5e6302fc32.
