# Supplementary material for: Integration of ground survey and remote sensing derived data: Producing robust indicators of habitat extent and condition
Source: Ecol Evol. 2019 Jun 20;9(14):8104–12. doi: 10.1002/ece3.5376 (PMC6662320; doi:10.1002/ece3.5376)
Supplement: Supplementary file 1 [file ECE3-9-8104-s001.docx]

**Appendix A**

Figures showing the spatial pattern of the bias in the LCM data represented by the $\alpha$ and $\beta$ terms in Equation 2 for each of the four broad habitats modelled.

Figure A.1 Maps showing estimated values for alpha and beta according to Equation (2) for Broadleaved Woodland

 Figure A.2 Maps showing estimated values for alpha and beta according to Equation (2) for Bog

Figure A.3 Maps showing estimated values for alpha and beta according to Equation (2) for Arable land

 Figure A.4 Maps showing estimated values for alpha and beta according to Equation (2) for Fen, Marsh and Swamp land
